# Outcomes of arthroscopic elbow synovectomy and neurolysis of the ulnar nerve for tenosynovial giant cell tumor in a young athlete: a case report and literature review

**DOI:** 10.1016/j.jseint.2023.07.003

**Published:** 2023-07-28

**Authors:** Osama Alzobi, Ghislain Aminake, Ayyoub Mohammed, Ashraf Hantouly, Theodorakys Marín, Bashir Zikria

**Affiliations:** a Aspetar Orthopaedic and Sports Medicine Hospital, Doha, Qatar; b Department of Orthopaedic Surgery, Surgical Specialty Center, Hamad Medical Corporation, Doha, Qatar

**Keywords:** Tenosynovial giant cell tumor, TGCT, Pigmented villonodular synovitis, PVNS, Elbow, Arthroscopy

Tenosynovial giant cell tumor (TGCT), previously known as pigmented villonodular synovitis, is a benign neoplastic condition that arises from the synovium, tendon sheath, and bursae of joints.^7^ It is characterized by the proliferation of abnormal giant cells and synovial tissue, which can cause joint effusion, pain, stiffness, bone erosion, and the accumulation of hemosiderin deposits.^7^

Chassaignac was credited as the first to describe TGCT at the flexor tendons of the middle and index fingers in 1852.^12^ Later, it was subdivided into diffuse and localized forms.^12^ Localized TGCT typically involves a discrete area of a joint, bursa, or tendon sheath. In contrast, diffuse TGCT involves the entire synovium of the joint and is often more aggressive and difficult to treat.^3^ TGCT is most commonly found in the knee joint. However, it is also possible for TGCT to occur in other joints, such as the hip, ankle, and shoulder joints. TGCT in the elbow is relatively rare, with only 37 cases reported in the literature.^1^^,^^3^^,^^9^^,^^11^ Treatment may include open or arthroscopic synovectomy, radiation therapy, or medication. Currently, very limited information exists about the arthroscopic treatment and outcomes after surgery for the elbow joint. In the case study, we present a 20-year-old male padel player with TGCT of the elbow who was treated with arthroscopic complete synovectomy and débridement with a two-year follow-up period.

The patient was informed that data concerning the case would be submitted for publication, and he provided consent.

## Case report

**Figure 1:**
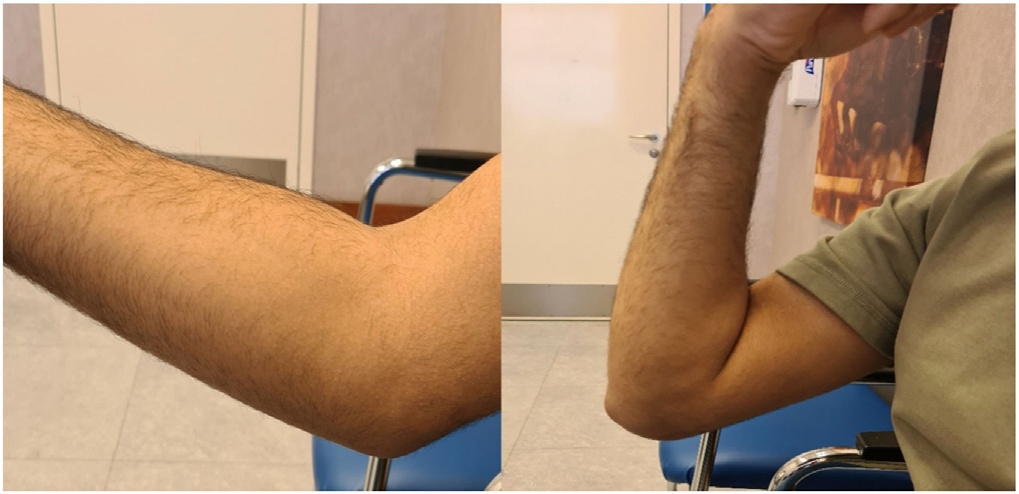
Preoperative range of motion of the elbow, demonstrating limited flexion and extension.

**Figure 2:**
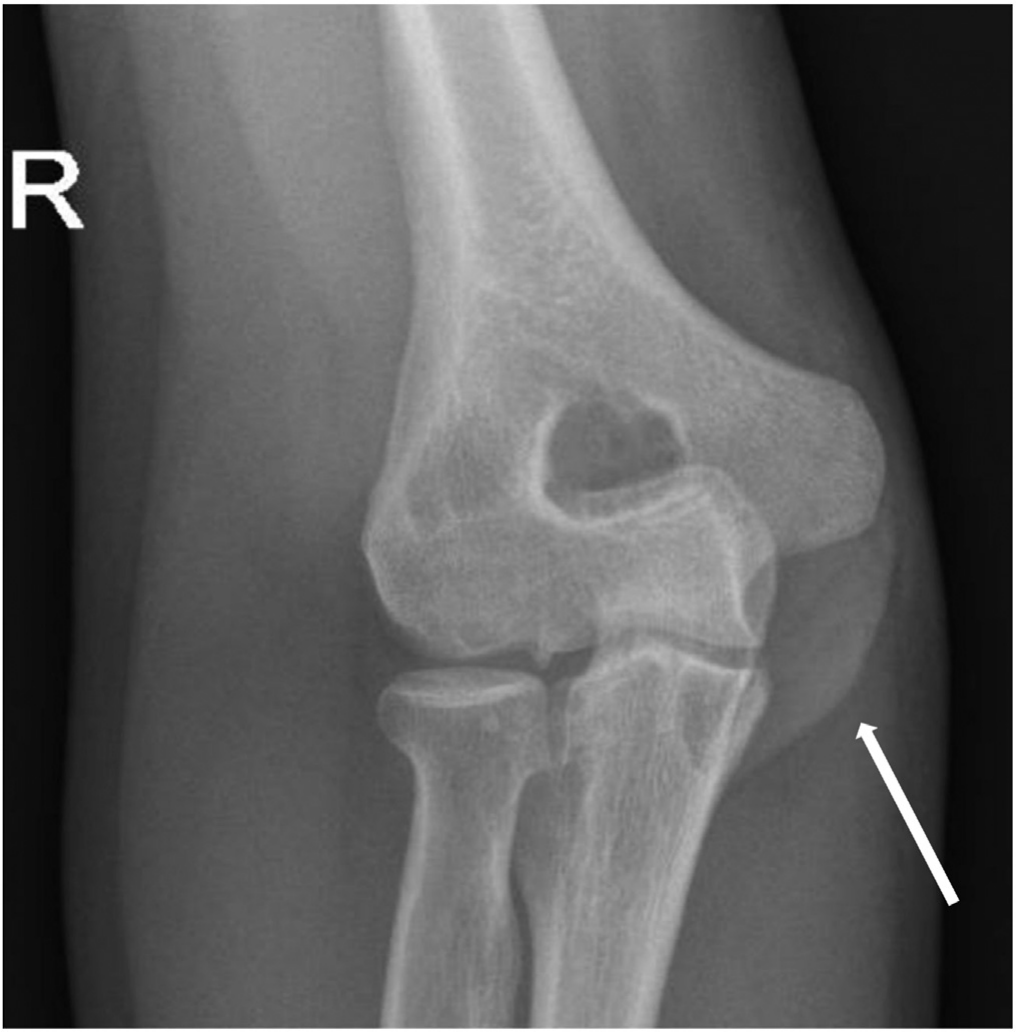
Radiograph showing a soft tissue mass (arrow) on the right elbow, without any evident of bone erosion.

**Figure 3:**
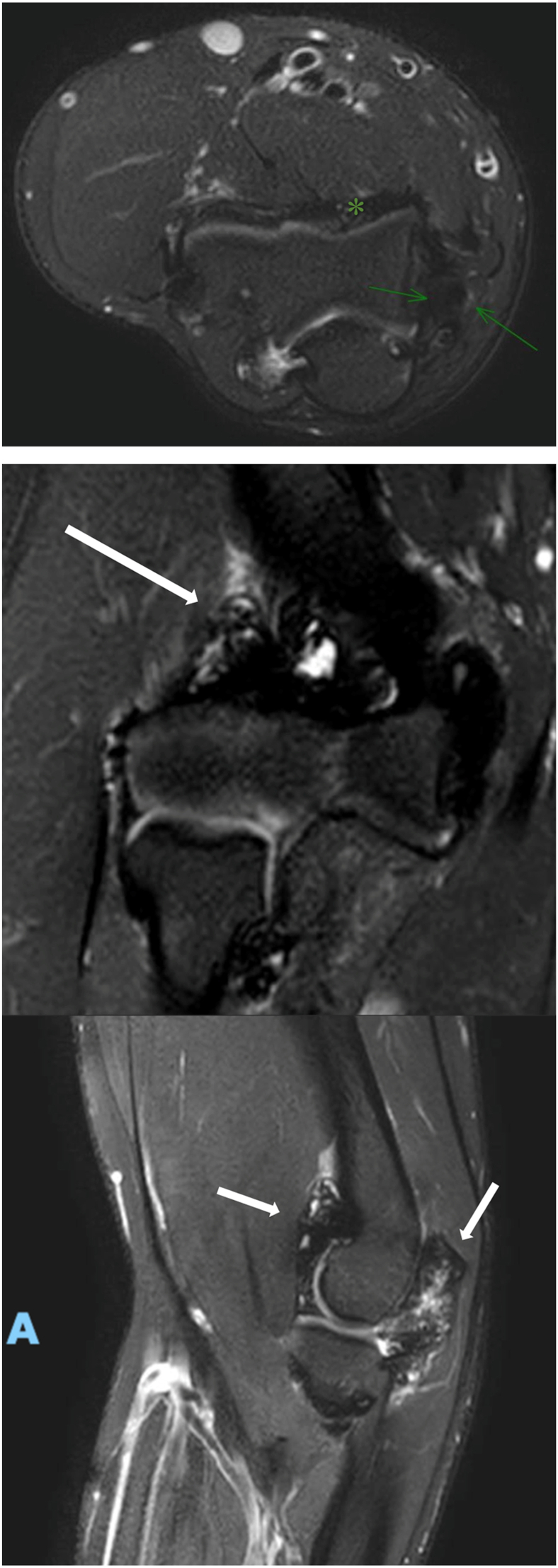
Preoperative MRI showing synovial thickening (∗), displacement of ulnar nerve (green arrows) and diffuse intra-articular nodular masses (white arrows). *MRI*, magnetic resonance imaging.

A 20-year-old male patient, right-hand dominant and a professional padel player, has been following up with a primary sports medicine physician for a year with a chief complaint of right elbow pain with a limited range of motion of 20 to 125 degrees, explicitly limited in extension. There was no history of trauma. A radiograph showed no significant bone-related issues. Of incidental note was a fenestrated olecranon fossa with wider margins. Ultrasound showed a joint effusion with chronic synovitis. The patient had improvement in pain and range of motion after undergoing physiotherapy for three months. One year later, he experienced a setback with loss of motion and increased pain to 3 of 10. He was referred for follow-up with a surgeon. During the appointment, the patient reported a painful elbow, accompanied by swelling and decreased range of motion (35 to 115 degrees actively) (Fig. 1). He also complained of paresthesia and ulnar nerve symptoms. His Elbow Mayo Score was 65. A radiograph of the right elbow revealed a soft tissue mass around the joint, with no apparent bone erosion or cystic changes (Fig. 2). A right elbow magnetic resonance imaging (MRI) demonstrated synovial thickening and diffuse intra-articular nodular masses suggestive of diffuse TGCT with extension of synovial thickening inside the cubital tunnel causing displacement of the ulnar nerve (Fig. 3). After discussing our findings, the risks of the surgery, and recurrence with the patient, the decision was made to proceed with elbow arthroscopy with synovectomy and neurolysis of the ulnar nerve.

**Figure 4:**
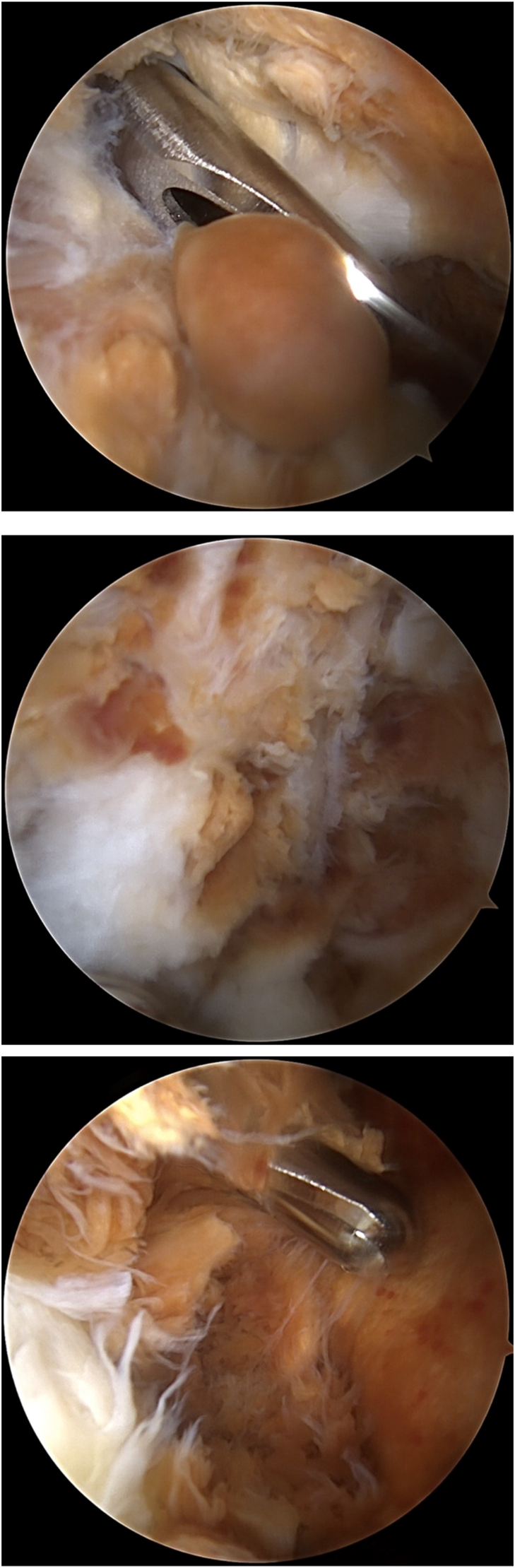
Arthroscopic findings, diffuse TGCT with nodular areas before synovectomy. *TGCT*, tenosynovial giant cell tumor.

**Figure 5:**
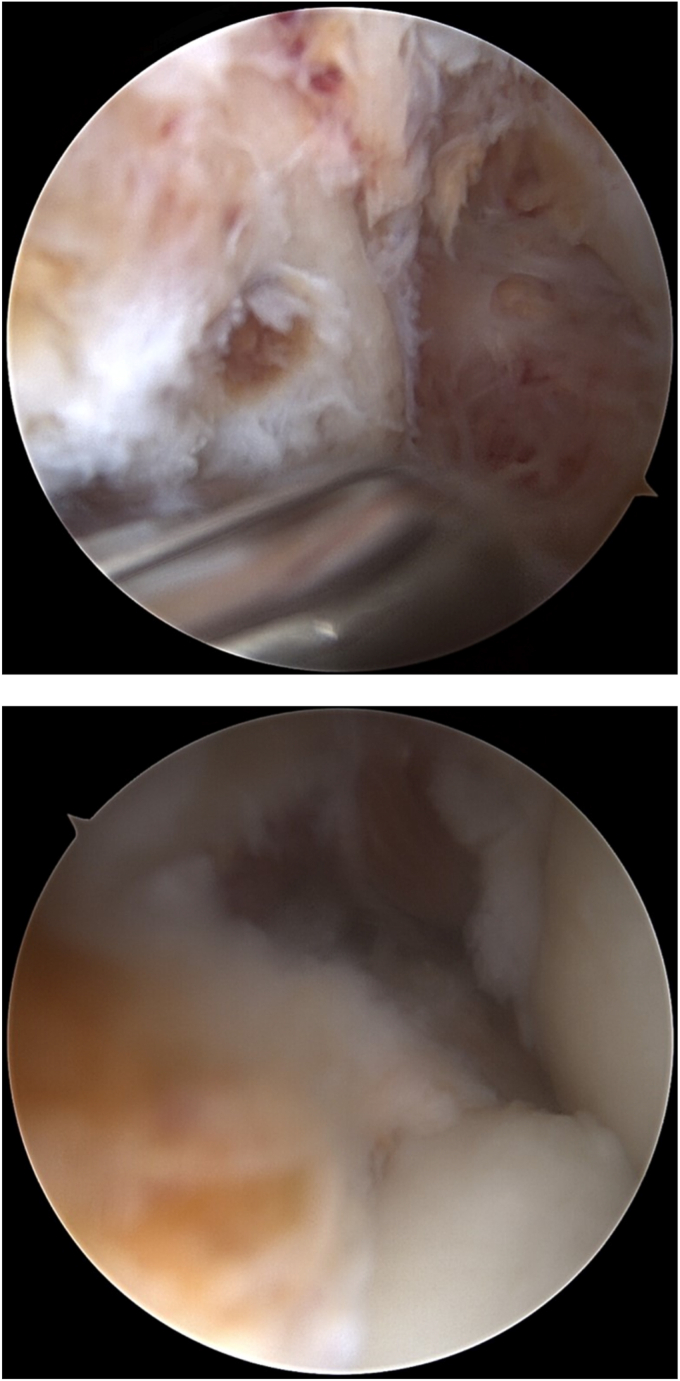
Arthroscopic findings after synovectomy.

Anesthesia was induced, and the patient was placed in the lateral decubitus position with an arm holder. The patient underwent a preoperative examination during which his elbow range of motion was measured between 30 and 120. Following this, the patient was prepared and draped in a sterile manner, and then neurolysis of the ulnar was performed with a small mini-incision. The nerve was stable, and the thickened tissue was excised in the cubital tunnel. There was a palpable elbow effusion; saline was injected through the soft spot, but less saline was needed because of the effusion. Arthroscopy was initiated through a standard proximal anteromedial portal, and a proximal lateral portal was made under direct visualization. The visualization in the anterior compartment was limited due to the diffuse TGCT with nodular areas (Fig. 4). The tissue was excised for biopsy. A shaver was introduced, and a complete synovectomy and débridement were performed (Fig. 5). There was Grade 2 and 3 erosion of the cartilage. The posterior compartment was visualized through a posterolateral portal and a trans-triceps central portal. Significant synovitis and several large nodules were excised. The posteromedial and posterolateral gutters were visualized and débrided as well. There was significant synovitis in the posterolateral gutter, so it was necessary to make a mid-lateral “soft spot” for better access to débride the diffuse synovitis. Before closing, the anterior compartment was visualized, and an anterior capsule release was performed. The arthroscope was removed, and manipulation under anesthesia was performed. The range of motion was from 0 to 135 degrees. The wounds were cleaned with copious irrigation and closed with nylon. A posterior splint was applied with the elbow in a neutral position.

**Figure 6:**
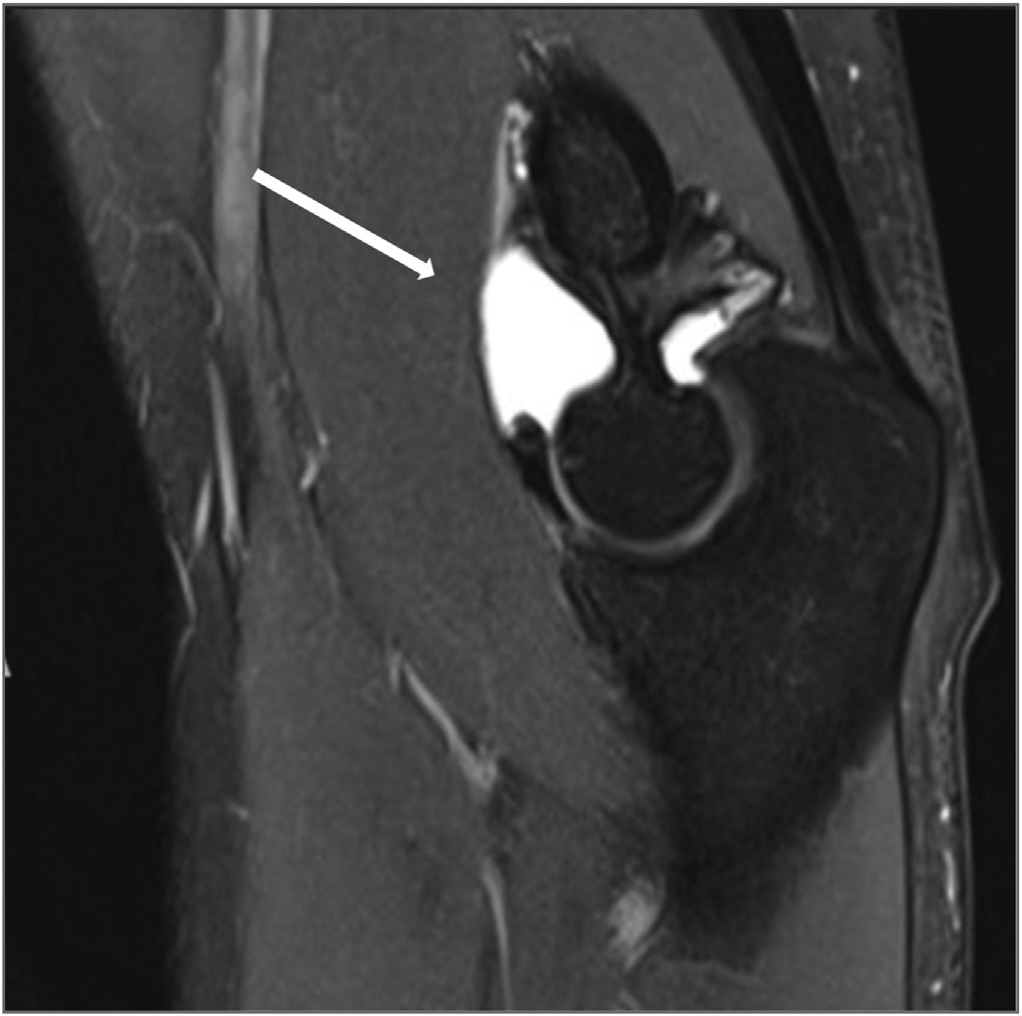
Postoperative MRI depicting clear elbow joint space (white arrow) and absence of synovial thickening. *MRI*, magnetic resonance imaging.

**Figure 7:**
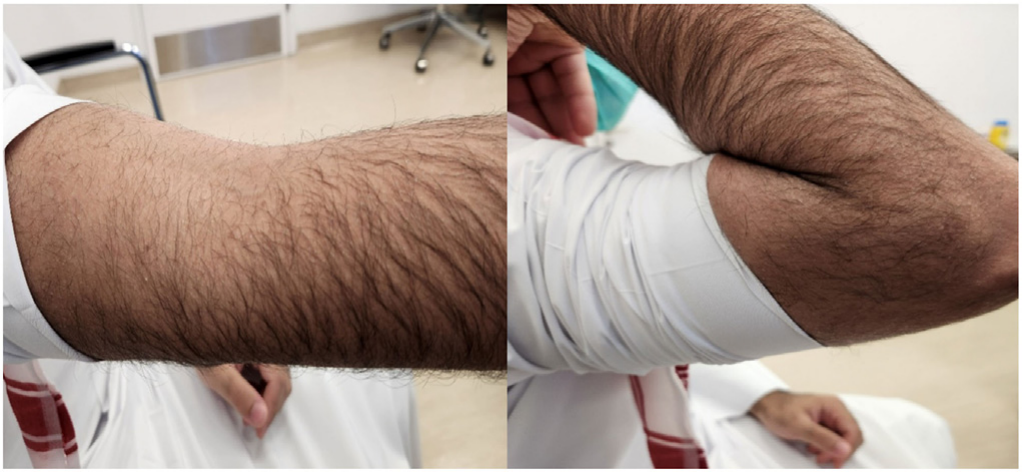
Postoperative elbow range of motion after two years.

Postoperative range of motion was started immediately as tolerated the next day after surgery. An MRI was done two years after the surgery and showed no remaining nodular masses in the joint cavity (Fig. 6). Histopathology results confirmed the suspected diagnosis of TGCT. At his 24-month visit, he had achieved zero degrees of extension with 130 degrees of flexion and a Mayo Elbow Score of 100 (Fig. 7).

## Discussion

Elbow arthroscopic surgery is a minimally invasive surgical procedure with relatively low complication rates.^2^ Common indications may include loose body extraction, contracture release, synovectomy, and lateral epicondylitis release. In addition, the indications have been extended to include more complex conditions such as radial head resection, arthroscopically assisted fixation of intra-articular fractures, total synovectomy, and ligament reconstruction.^2^^,^^3^^,^^10^

While TGCT is not considered a malignant tumor, it can still cause significant damage to the affected joint and nearby tissues, including bone and cartilage, leading to severe joint destruction, deformity, and even amputation if left untreated.^11^ In addition, TGCT has a high potential for recurrence, even after surgical removal.^3^ Due to the low incidence of TGCT, it can be challenging to establish a universal standard for treatment. Total synovectomy is the current consensus for treating such lesions in the knee, hip, shoulder, and elbow joints for either localized or diffused TGCT.^3^^,^^9^ However, until now, there has been no clear consensus regarding the most appropriate surgical synovectomy, either with an arthroscopic or open approach. Regardless of the technique, the goal is to obtain sufficient removal of the lesion necessary to reduce the possibility of recurrence.^5^ Open synovectomy has been associated with complications such as infection risk, suture dehiscence, joint stiffness, and postoperative pain ^6^ An arthroscopic synovectomy is minimally invasive and has been found to have fewer complications and a faster postoperative recovery time.^2^^,^^6^

Few reports have investigated arthroscopic and open management of TGCT for localized and diffuse disease in the elbow joint. Al-Farii et al^3^ reported that the recurrence rate following open synovectomy is 17.4% in 4 out of 23 cases. It is important to note that this rate is similar to the recurrence rates reported in other studies on open synovectomy for diffuse TGCT of the knee, which vary from 8.0% to 22.6%.^6^ Additionally, arthroscopic techniques have been reported in a total of 14 patients in seven reports,^1^^,^^5^^,^^8^^,^^9^^,^^13^^,^^14^ with a recurrence rate of 14.3% reported in 2 out of 14 cases (Table I). The follow-up period for these cases ranged from 6 to 104 months. Lavignac et al^9^ reported that two out of eight patients required revision surgery with an open procedure due to a recurrence detected 6 and 21 months after arthroscopic total synovectomy. The authors acknowledged that the recurrence rates might be influenced by the technical challenges of posterior arthroscopic synovectomy and highlighted the importance of adequate follow-up to detect any recurrence.^9^ Koca et al^8^ described a similar arthroscopic technique for the treatment of TGCT in the elbow joint, but with the addition of adjuvant radiotherapy as a potential treatment option to improve local control and minimize the risk of recurrence. The authors reported no recurrence after 20 months of follow-up. However, the effect of adjuvant radiotherapy is still uncertain because there are risks of serious complications, including skin necrosis and potential malignant transformation.^3^ Pexidartinib, the first oral medication for TGCT approved by the US Food and Drug Administration, functions as a colony-stimulating factor-1 receptor antagonist. Nevertheless, it carries a risk of severe liver damage. In a study conducted by Bernthal et al,^4^ recurrent diffuse TGCT was observed in three patients, affecting the hip, knee, and foot. The authors reported a combined treatment approach involving surgery and preoperative pexidartinib to minimize the likelihood of disease recurrence after the operation. The study demonstrated enhancements in pain relief and local disease control.

**Table I:** This table summarized the clinical results of elbow arthroscopic synovectomy.

Study	Year	N	Age (y)	Gender	Type	Adjuvant treatment	Position	Protals (n)	FU (mo)	Recurrence
Ekman et al^5^	1997	1	57	M	Diffuse	Non	Prone	NS	72	No
Su et al^14^	2008	1	8	F	Diffuse	Non	NS	4	NS	No
Koca et al^8^	2012	1	41	F	Diffuse	Radiosynovectomy	NS	NS	20	No
Ramos et al^13^	2016	1	43	M	Diffuse	Non	Prone	4	12	No
Afana et al^1^	2022	1	36	M	Diffuse	Non	Prone	4	6	No
Lavignac et al^9^	2022	8	41	F	Localized	Denosumab	Lateral decubitus	NS (5-7)	54	No
			35	M	Localized	Denosumab			83	No
			38	F	Diffuse	Denosumab			72	No
			31	M	Diffuse	Bisphosphonate			104	Posterior
			44	F	Localized	Ulnar nerve release			42	No
			48	M	Diffuse	NSAID, Denosumab			51	No
			59	F	Diffuse	Denosumab			98	No
			54	F	Diffuse	Denosumab, Ulnar nerve release			27	Posterolateral
Alzobi et al	2023	1	20	M	Diffuse	Ulnar nerve release	Lateral decubitus	7	24	No

*NSAID*, non-steroidal anti-inflammatory drugs.

Based on this review, arthroscopic total synovectomy was found to be a comparably effective treatment option as the open technique, but with a reduced rate of recurrence when treating TGCT in the elbow. However, the current evidence regarding the superiority of open or arthroscopic techniques in minimizing the risk of recurrence is not yet robust enough to provide a definitive answer. More studies with larger sample sizes are needed to establish which technique is better for TGCT treatment in the elbow joint.

## Conclusion

The arthroscopic technique used in this case report offers a minimally invasive, viable alternative to open synovectomy and has shown promising clinical outcomes with no recurrence after two years.

## Disclaimers

Funding: No funding was disclosed by the authors.

Conflicts of interest: The authors, their immediate families, and any research foundation with which they are affiliated have not received any financial payments or other benefits from any commercial entity related to the subject of this article.

Patient consent: The authors confirm they have obtained patient informed consent form(s) for this case report.
